# Breaking the cycle: Reforming pesticide regulation to protect pollinators

**DOI:** 10.1093/biosci/biad088

**Published:** 2023-10-23

**Authors:** Adrian Fisher, Rafaela Tadei, May Berenbaum, James Nieh, Harry Siviter, James Crall, Jordan R Glass, Felicity Muth, Ling-Hsiu Liao, Kirsten Traynor, Nicole DesJardins, Roberta Nocelli, Noa Simon-Delso, Jon F Harrison

**Affiliations:** School of Life Sciences at Arizona State University, Tempe, Arizona, United States; São Paulo State University, Rio Claro, Brazil; University of Illinois at Urbana-Champaign, Urbana, Illinois, United States; University of California, San Diego, California, United States; University of Texas at Austin, Austin, Texas, United States; University of Bristol, Bristol, England, United Kingdom; University of Wisconsin-Madison, Madison, Widsconsin, United States; School of Life Sciences at Arizona State University, Tempe, Arizona, United States; University of Texas at Austin, Austin, Texas, United States; University of Illinois at Urbana-Champaign, Urbana, Illinois, United States; University of Hohenheim, Stuttgart, Germany; School of Life Sciences at Arizona State University, Tempe, Arizona, United States; Federal University of São Carlos, Araras, Brazil; BeeLife European Beekeeping Coordination, Louvain la Neuve, Belgium; School of Life Sciences at Arizona State University, Tempe, Arizona, United States

**Keywords:** pollinators, pesticides, policy, regulation, sustainability

## Abstract

Over decades, pesticide regulations have cycled between approval and implementation, followed by the discovery of negative effects on nontarget organisms that result in new regulations, pesticides, and harmful effects. This relentless pattern undermines the capacity to protect the environment from pesticide hazards and frustrates end users that need pest management tools. Wild pollinating insects are in decline, and managed pollinators such as honey bees are experiencing excessive losses, which threatens sustainable food security and ecosystem function. An increasing number of studies demonstrate the negative effects of field-realistic exposure to pesticides on pollinator health and fitness, which contribute to pollinator declines. Current pesticide approval processes, although they are superior to past practices, clearly continue to fail to protect pollinator health. In the present article, we provide a conceptual framework to reform cyclical pesticide approval processes and better protect pollinators.

Trevan (1927) proposed the median lethal dose (LD_50_) as one useful measure for standardizing drug dosages, but in the intervening 95 years, it has become the foundation for assessing the acute toxicity and environmental safety of agrochemicals, including pesticides. But there is almost a century of evidence that the LD_50_ metric has systematically failed to identify critical negative impacts (just short of lethality) on beneficial insects that provide important ecosystem services—most notably, pollination. Reliance on lethality assessments has created a repeating cycle in which pesticides are approved and used until detrimental impacts on nontarget species are discovered, with ensuing restrictions, followed by the development of new replacement pesticides that are often subsequently discovered to also be hazardous (figure 1). Many studies have demonstrated that numerous approved pesticides have significant negative sublethal effects on the behavior, physiology, and reproduction of managed and wild pollinators, both of which are economic contributors to agriculture, at field-realistic levels (Desnaux et al. 2007, Siviter et al. 2021a, Tosi et al. 2022). In addition, because biological processes tend to be conserved within a taxon, the documentation of harmful sublethal effects of approved pesticides on managed or model pollinators almost certainly means that the pesticides are also dangerous for nonmodel, nonmanaged pollinators (Woodcock et al. 2016).

**Figure 1.**
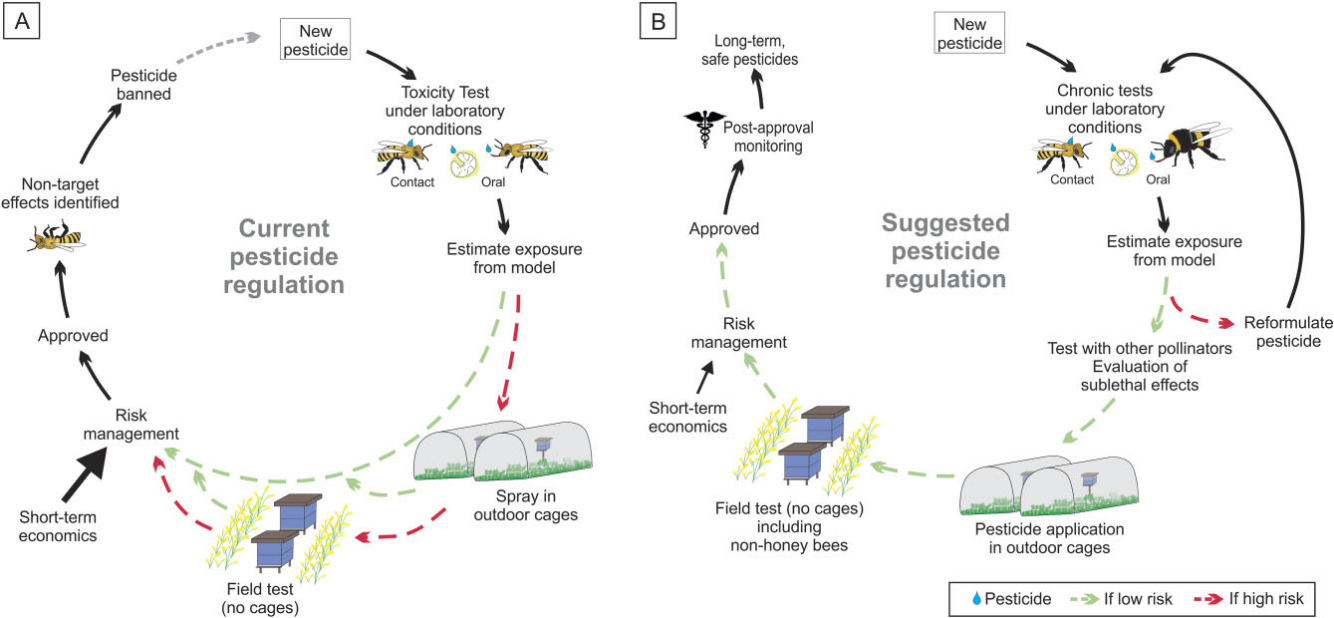
Comparison of current pesticide regulatory practices and a suggested model for improvement. (a) The current pesticide assessment process includes approval of a pesticide On the basis of low mortality of larvae and caged honey bee adults in contact and oral exposure tests. The current practices allow bypassing of spraying in outdoor cages and field tests (no cages) instead using only first tier tests to indicate low risk On the basis of short-term LD_50_ evaluations. In addition, current practices in most countries do not include postapproval monitoring that could result in the banning of a pesticide because of its nontarget effects. (b) The suggested improved pesticide approval and monitoring process includes requirements for multiple levels of testing with field- and taxon-relevant exposure conditions and risk assessment before a pesticide is approved. This process would also require assessing pesticide toxicity for diverse pollinator species, testing of sublethal effects, and synergistic interactions between pesticides. In addition, our proposed model includes post-approval monitoring allowing for enhanced efficiency in detecting unanticipated negative effects.

Many wild pollinator species are experiencing population declines, whereas managed pollinators, most notably honey bees (*Apis mellifera*), are experiencing economically unsustainable regional losses, threatening sustainable food security, ecosystem function, and human health (Smith et al. 2015, Reilly et al. 2020). Although the relative importance of pesticides to insect decline remains unclear, there is unequivocal evidence that pollinator safety across many landscapes is put at risk by current pesticide use practices (Siviter et al. 2021b). Long-term preservation of both biodiversity and agricultural sustainability demands a major revision of pesticide approval systems (Drivdal and van der Sluijs 2021). In the present article, we suggest a pathway to develop pesticide approval systems to anticipate and prevent inadvertent adverse impacts of pesticides on nontarget species. We first highlight the limitations of current pesticide regulation protocols and then suggest steps for improving pesticide regulation, including harnessing recent technological advances that overcome the limitations of the current regulation system.

## Current regulatory approaches and inadequacies

Pesticide toxicity is currently tested with a tiered approach that is intended to allow approval of only those chemicals at low risk of causing adverse impacts on the biotic environment (EPA 2014a, EFSA et al. 2023). The risks presented by pesticides to pollinators that encounter them have long been assessed using honey bees as a proxy for the thousands of insect species that contribute to plant pollination. In the United States and Brazil, exposures are estimated with the Bee-REX model, which incorporates worst-case scenario exposure events (EPA 2014b, Cham et al. 2020). In Europe, various exposure models have been proposed depending on the pesticide application way (i.e., contact and dietary models), leading to various predictive exposure concentrations that result from the consideration of parameters such as the application rate, exposure factors, the body surface factor, or the food consumption (EFSA et al. 2023). These are then compared with LD_50_ values under laboratory conditions. Many pesticides undergo only a first-tier screen with honey bee adults and larvae under laboratory conditions. Next-tier studies can include quantification of pesticide residues in pollen and nectar collected by bees and pesticide effects on physiology and behavior of lab-reared bees. These next-tier studies are conducted only if the calculated risk quotients from the laboratory studies exceed the levels of concern or if other data indicate behavioral or fitness consequences of the pesticide during the approval process (CFR40C § 158.630; EPA 1996). Similarly, the highest tier studies of effects of pesticides on pollinators in the field are conducted only if concerns arise from lower tier studies (European Commission 2011, EPA 2014a, Sanchez-Bayo and Goka 2014). The effects of pesticide toxicity have become more apparent over time because of development of methodologies focusing on more subtle observations than mortality. This suggests that current exposure duration standards for tier 1 tests do not allow for assessments of chronic nor sublethal effects to clearly lay out proper boundaries for protecting pollinators (Sanchez-Bayo and Tennekes 2020).

Unfortunately, this system has repeatedly failed to identify sublethal injurious effects on pollinators. Field-realistic concentrations of numerous approved fungicides, herbicides, and insecticides demonstrably compromise pollinator health (Iwasaki and Hogendoorn 2021). At the individual level, exposure to certain pesticides can undermine learning and navigation abilities, digestive functioning, brood care, flight performance, and longevity (Tosi et al. 2017, Siviter et al. 2018, Kenna et al. 2019, Fisher et al. 2021, Fischer et al. 2023). At the colony level, sublethal consequences of field-realistic pesticide exposures include disruption of thermoregulatory capacity, reduction in colony size, suppressed male and female reproduction, increased brood disease, and reduced overwintering survival (Whitehorn et al. 2012, Crall et al. 2018, Traynor et al. 2021). Although the majority of such studies have been performed with honey bees as the model nontarget species, bumble bees, stingless bees, and solitary bees can experience comparable or even more severe adverse impacts (Artz and Pitts-Singer 2015, Crall et al. 2018).

Failures to identify and document the adverse ecological impacts of pesticides arise in multiple ways. One clear type of failure has been the reliance on laboratory lethality (i.e., LD_50_) assays for short exposures (European Commission 2013). Short-term LD_50_s are problematic because pollinators generally experience exposure to agrochemicals for much longer than a few days, and the toxicities of agrochemicals to pollinators have been well-demonstrated to increase with duration of exposure (Simon-Delso et al. 2018, Sánchez-Bayo and Tennekes 2020). Also, LD_50_s characterize a dose that is highly toxic, killing 50% of the population, and the relationships between such a dose and the dose that will have a minimal effect on pollinators are generally poorly characterized. Current approval procedures do not as a matter of course include assessments of sublethal effects or field-realistic outcomes (European Commission 2002, EPA 2014a). Characterizing the links between sublethal effects on individual performance (e.g., learning and navigation abilities, digestive functioning, and thermoregulation), pollinator fitness typically requires long assessment periods under field-realistic exposure regimes. For eusocial pollinators, such as honey bees and bumble bees, toxicity tests on individual workers, most of which are sterile (nonreproductive), do not predict colony-level effects mediated by the responses of the few reproductive individuals in the colony to pesticide exposure or by colony-level feedback regulation. Another limitation of current procedures is the narrow focus on isolated active ingredients, whereas, in reality, many pesticide formulations contain mixtures of chemicals that can interact with other factors or contain “inert ingredients” that are, in fact, toxic (Straw et al. 2022). Moreover, pollinators may be exposed simultaneously to diverse types of pesticides, as well as to secondary stressors (e.g., disease, poor nutrition) that may sometimes have strong interactive or additive effects with pesticides in the field.

The near-exclusive reliance on honey bees as a model species in toxicity testing is problematic, as has been demonstrated by multiple direct sensitivity comparisons across bee species (EFSA Panel on Plant Protection Products and their Residues 2012, Arena and Sgolastra 2014, Gradish et al. 2019). Pollinators differ widely in life history and physiology, resulting in tremendous variation in exposure routes and susceptibilities (Hladik et al. 2016, Boyle et al. 2019, Gradish et al. 2019). Finally, challenges in estimating the persistence of pesticides in the environment have caused insufficient estimations of exposure (Traynor et al. 2021). As a prime example, exposure to wind-blown dust from seeds coated with neonicotinoids was not predicted to be a major route of exposure when these pesticides were approved (Sgolastra et al. 2020, Greatti et al. 2003).

## Solutions

Over the past century of pesticide testing, improvements have been made in better describing the routes of pesticide exposure and developing methodologies to reveal their effects. That said, maintaining such a slow pace of advancement is insufficient for the task of protecting pollinators. We offer a five-part solution:

### Thorough laboratory screening

The current reliance on mortality levels (i.e., LD_50_) within a short time frame (48 hours) as the standard for assessing pesticide toxicity to insect pollinators is the first limitation in adjusting procedural inadequacies (OECD 1998). For other model organisms, LD_50_ testing has been discouraged and replaced with other methods that monitor lethality and various outcomes besides mortality for animal subjects (Erhirhie et al. 2018). Recently, the European Food Safety Authority (EFSA) reconsidered the hazard parameters in experimental studies and proposed to extract more information from the toxicological tests, considering the whole dose–response curves (from which other toxicological endpoints could be extrapolated—no observed effect concentration or LD*x*—calculating the existence of time-reinforced toxicity or integrating sublethal effects and mixture toxicity; EFSA et al. 2023). Such an approach with pollinators would increase test sensitivity and aid in addressing the disconnection between laboratory and field results where outcomes of exposure include a variety of sublethal effects at low concentrations. In addition, the current limits for oral toxicity assessments range from 48 hours to 10 days, which doed not reflect field-relevant exposure or allow for adequate evaluation of resulting outcomes (OECD 1998, OECD 2017, Simon-Delso et al. 2018). Assessments of mortality should discard rigid timeframes, instead allowing the lifespan and natural history of test organisms or the persistence of pesticide residues in nature to factor into exposure and overall test duration (Tosi et al. 2021). In general, toxicological testing for risk assessment purposes should be prolonged in time. Furthermore, decision-makers should consider the methodological developments proposed by the EFSA (EFSA et al. 2022, 2023), which may allow for progress in describing the pesticide toxicological profile and its risks to pollinators. On the basis of this information, decision-makers will be able to base their approval decisions on more relevant information.

### Relevant sublethal testing

The approval process must include assessments of sublethal effects on individuals and colony-level effects such as reproduction under a range of possible concentrations and realistic exposure times for pollinators. Adjusted toxicity standards using LD_10_ values or NOEL (no observed effect levels) would allow for reduced lethality and more careful discernment of nonlethal detrimental effects of exposure to establish protective standards. Studies that include measurement of pesticide concentrations in resources collected and used by pollinators are needed for calculating field-realistic exposures (Linguadoca et al. 2021). For eusocial pollinators, colony-level testing must be mandatory to determine safe-use levels because effects on colony reproduction or colony-level behavioral interactions cannot be assessed fully by testing individuals in the laboratory. In addition, prolonged laboratory tests using field-realistic concentration and exposure time can be useful to show physiological, behavioral, and other sublethal effects as a proxy for determining the effects on pollinator population growth and development (Simon-Delso et al. 2018). Also, tests targeted at uncovering underlying mechanistic effects of exposure can inform physiological outcomes and behavioral observations. Methods for measuring sublethal or colony-level effects that are not prohibitively expensive are available and well-established for many taxa. Technological advances are making scalable, cost-effective studies of the sublethal and synergistic impacts of agrochemicals feasible. In particular, the rapid emergence of computer vision tools for behavior provides new opportunities for developing reliable, standardized, and economical screening protocols (Crall et al. 2018, Høye et al. 2020).

### Testing diverse species

In view of the physiological and ecological diversity that exists across pollinator taxa, testing procedures must include regionally appropriate managed and native bees and other pollinator species likely to be exposed to the pesticide. In many tropical regions, stingless bees are primary pollinators, and they have been shown to respond differently to multiple pesticides than honey bees (Cham et al. 2018). Similarly, multiple native bee species have different life histories than honey bees, including soil nesting and varying levels of sociality and, therefore, experience differing exposure routes and risks (Boyle et al. 2019, Franklin and Raine 2019, Sgolastra et al. 2019). The approval process must assess the extent to which native pollinators are likely to experience pesticide exposure, so that toxicity levels can be measured with field-realistic exposures. In the revised guidance to pollinator risk assessment from the EFSA, at least one species of bumble bee and one species of solitary bee have been proposed for inclusion in the pesticide regulation (EFSA et al. 2023). However, a number of other insect pollinators with differing biology and vulnerability to pesticides may exist, which should, ideally, be considered in the future improvement of the pesticide risk assessment (EFSA et al. 2022). In addition, nonbee insect pollinators provide substantial contributions to pollination, can experience similar agrochemical exposure, and should be considered for toxicity testing (Rader et al. 2015, Uhl and Brühl 2019).

### Mandatory field-realistic testing

The regulatory community should implement and mandate thorough and cost-effective safety testing mechanisms to ensure that approved pesticides are safe for pollinators under realistic environmental conditions. Although they are few in number, several field tests of pesticide exposure demonstrate numerous negative outcomes for multiple pollinator species (Rundlöff et al. 2015, Tsvetkov et al. 2017, Woodcock et al. 2017, Olaya-Arenas et al. 2020, Fisher et al. 2022). The repeated failures of the current system for predicting nontarget pesticide toxicity demonstrate that laboratory determinations of LD_50_ values are insufficient. Conceivably, improvements in lower-tier testing as described above might ameliorate the need for field testing. However, given that environmental conditions usually differ dramatically from lab conditions, that environmental conditions such as temperature affect toxicity (Kenna et al. 2023), and that for eusocial insects, colony-level testing is critical to assess effects on reproduction, mandating higher-tier field-realistic testing before approval decisions are made could significantly lower threats to pollinator safety (figure 1). In addition, pesticides should be tested as they are formulated and sold for real-world use to account for the potential effects of inert ingredients and the synergisms between formulation components. Field tests can improve the interpretation of results in combination with the lethal and sublethal tests proposed in the present article, their results being considered as complementary to the laboratory tests rather than as probatory of risk or no risk (figure 1b)

### Postapproval monitoring (fitopharmacovigilance) and reassessment

It is difficult for scientists to anticipate all important field fates of pesticides and their interactions, so postapproval monitoring and reassessment of provisionally approved pesticides, as is routinely performed for pharmaceuticals (Milner and Boyd 2017), must be implemented. This process should include surveys of both managed and unmanaged pollinators exposed to pesticides applied in the approved manner (figure 1).

Developing an effective system for testing pesticide safety will require the engagement of many more stakeholders, including those with expertise in chemical environmental degradation and pesticide residues in realistic environmental conditions, as well as those with expertise in pollinator behavior, physiology, and natural history. A layered approach with multiple testing levels accounting for underlying mechanisms paired with clear measures of behavior and fitness will provide policymakers with the best data for making pesticide approval decisions (Ankley et al. 2010, Sponsler et al. 2019). Overhauling the current pesticide approval system will require significant political and economic resolve. Recently, the United States Geological Survey (USGS) unveiled plans to reduce the extent of their pesticide monitoring and frequency of pesticide use reporting as part of the Pesticide National Synthesis Project (USGS 2023). Such measures follow prior reductions in the last decade that also scaled back the comprehensiveness of the USGS's pesticide monitoring program (Hitaj et al. 2020, Douglas 2023). Although it was once a valuable tool for researchers, this reconfiguration of the USGS pesticide database constitutes a major setback for investigations into pesticide use and pollinator declines (Gewin 2023). Legislative input in maintaining pesticide monitoring databases and implementing stringent postapproval pesticide evaluations is therefore essential for enhanced pollinator protection. The consequences of reduced pesticide monitoring and inaction in changing toxicity testing could have wide spread ramifications, especially if the losses of managed or wild pollinators increase. Pollinator insufficiency could compromise food security for vulnerable populations around the world (Steffan-Dewenter et al. 2005).
